# In-Plane Behaviour of Masonry Walls: Numerical Analysis and Design Formulations

**DOI:** 10.3390/ma14195780

**Published:** 2021-10-03

**Authors:** Thomas Celano, Luca Umberto Argiento, Francesca Ceroni, Claudia Casapulla

**Affiliations:** 1 Department of Engineering, University of Naples Parthenope, Centro Direzionale Is. C4, 80143 Napoli, Italy; thomas.celano@uniparthenope.it; 2 Department of Structure for Engineering and Architecture, University of Naples Federico II, Via Forno Vecchio, 80134 Napoli, Italy; lucaumberto.argiento@unina.it (L.U.A.); casacla@unina.it (C.C.)

**Keywords:** masonry walls, finite element model, discrete element model, in-plane behaviour, shear resistance, flexural resistance, ductility, design formulations

## Abstract

This paper presents the results of several numerical analyses aimed at investigating the in-plane resistance of masonry walls by means of two modelling approaches: a finite element model (FEM) and a discrete macro-element model (DMEM). Non-linear analyses are developed, in both cases, by changing the mechanical properties of masonry (compressive and tensile strengths, fracture energy in compression and tension, shear strength) and the value of the vertical compression stress applied on the walls. The reliability of both numerical models is firstly checked by means of comparisons with experimental tests available in the literature. The analyses show that the numerical results provided by the two modelling approaches are in good agreement, in terms of both failure loads and modes, while some differences are observed in their load-displacement curves, especially in the non-linear field. Finally, the numerical in-plane resistances are compared with the theoretical formulations provided by the Italian building code for both flexural and shear failure modes and an amendment for the shape factor ‘*b*’ introduced in the code formulation for squat walls is proposed.

## 1. Introduction

Unreinforced masonry structures are the most common existing constructions in the Mediterranean area, thanks to a long history of natural stones as building materials, which are highly availability and easy to use. Overall, historical masonry constructions are still widely used throughout the world, often even as strategic buildings, and most of them require adequate prevention against seismic actions. Masonry structures are outstanding systems when subjected to compressive stresses, while, under lateral forces such as seismic ones, the low tensile strength of these materials generally leads to local or global failure modes, the latter related to shear or flexural mechanisms discussed in detail below.

In general, local failures mean the activation of out-of-plane mechanisms involving masonry elements that are exposed to seismic actions orthogonally to their plane. Several studies [1,2,3,4,5,6,7,8,9,10] and experiences from past earthquakes have shown that the vulnerability of masonry buildings to out-of-plane mechanisms is emphasized by the lacking or weakness of connections between elements (i.e., between walls or between walls and horizontal diaphragms). On the other hand, global failure modes only occur in masonry buildings when the in-plane strengths of their earthquake-resistant elements can be activated, i.e., when the connections are able to guarantee a ‘box’ behaviour for the whole building such that seismic actions are, thus, mainly transferred to the walls parallel to each other.

The vulnerability of masonry elements subjected to in-plane actions depends on the possible failure mechanisms, which are influenced by both their mechanical properties and geometrical parameters. Flexural failures are due to compressive or tensile strength along the end sections of the wall, while shear failures can occur differently, according to the type of masonry. In ‘regular’ masonry walls, indeed, shear failures are mainly related to sliding phenomena along the mortar-unit interfaces, which can occur along crack lines following horizontal joints or diagonally stepped lines involving both vertical and horizontal joints. Conversely, in the case of irregular/rubble masonry walls, shear failures are, in general, related to the tensile strength of the masonry along diagonal cracks, crossing both masonry units and mortar joints. In the particular case of chaotic masonry, the interlocking between the units avoids the activation of sliding phenomena along the mortar joints, since they are not systematic or well organized, leading to diagonal shear failure. A detailed discussion of the reliability of the literature and the code formulations surrounding the prediction of the in-plane shear resistance of masonry walls can be found in [11].

The in-plane capacity of masonry walls has been investigated in several experimental and numerical studies [11,12,13,14,15,16,17,18,19,20,21,22,23] that have analysed the influence of different parameters, such as: (a) mechanical properties of masonry; (b) constraint conditions; (c) vertical compression levels; (d) slenderness of the wall; and (e) masonry texture. Their mechanical properties directly influence both the maximum capacity and ductility of masonry walls, while their constraint conditions and slenderness drive their failure modes toward shear or flexural mechanisms. In the case of low compression levels, a sliding shear failure is generally favoured in regular masonry walls, while flexural and diagonal shear failures may occur for medium–high levels of compression. Finally, as previously cited, masonry texture can affect the type of shear failure (diagonal or sliding shear).

Several modelling strategies can be found in the literature regarding the most suitable description of masonry behaviour [24]. Particularly for finite element models, the three following approaches are mainly used: (1) detailed micro-modelling, (2) simplified micro-modelling, and (3) macro-modelling. In the detailed micro-modelling approach, the units and the mortar are modelled separately, while in the simplified micro-modelling approach the mortar is not directly modelled, but rather interface elements are used to model the interaction, i.e., the bond behaviour between mortar and units. Finally, the macro-modelling approach assumes the masonry as a continuous, homogeneous and isotropic material, without considering the real configuration of the units. Generally, the first two approaches are used to identify, in detail, the behaviour of regular masonry walls, while the third is more suitable for irregular/rubble masonry structures. Nevertheless, the macro-modelling approach is the most-used modelling strategy in the literature to assess the seismic behaviour of masonry structures, both when simulations regard whole structures and local behaviours [7,25], because it requires the assessment of very few parameters. Conversely, macro-modelling approaches based on the finite element method are not able to predict failure modes related to sliding mechanisms along unit-mortar joints, which need the use of the micro-modelling approach and the assessment of suitable interface-constitutive constraints. Thus, despite the wide range of literature on this topic, there is still some uncertainty about the applicability of macro-modelling approaches to predict the actual failure modes of regular masonry walls, particularly the shear failure, and about the main parameters involved. The current Italian code [26,27] indicates, indeed, that the formulation for the diagonal shear failure of irregular masonry walls can be adopted in favour of safety for regular masonry as well, but there is no literature highlighting to what extent this is acceptable.

To fill such a gap of information, this paper presents several parametric analyses aimed to identify the most influencing parameters on the in-plane response of slender and squat regular masonry walls, also by means of comparisons with code formulations. Two macro-modelling approaches are adopted: a finite element model (FEM), implemented in the Diana FEA software [28], and a discrete macro-element model (DMEM), introduced in the 3DMacro software [29]. The FEM simulates masonry as a homogenous material characterized by a non-linear response, and uses a detailed mesh refinement [30]. Conversely, the DMEM proposed by [31] considers masonry structures as assemblages of macro-elements that collapse through different failure mechanisms; such a discretization approach allows assessing the non-linear responses of masonry structures, with a limited computational effort and a low number of input parameters. Thus, the latter is also suitable for standard engineering design practice or for massive structures, for which the computational effort of a detailed FEM could become excessive.

The main advantage of the FE model is to provide detailed information on the evolution of crack patterns in masonry panels during their loading process, thanks to the reduced size of its mesh. This confirms that the choice of the FE model should be preferred if the purpose is the investigation of the local behaviour (i.e., the stress-strain state into a structural element), while the DME model is preferable for global-level analysis (i.e., for defining the capacity curves of structural elements or of whole structures). In fact, the DMEM requires lower computational and modelling effort than FE, without losing reliability in the results. Therefore, another purpose of this paper is to better emphasize the differences in the results provided by the two approaches and to demonstrate the reliability of the simpler DME model, which should be more convenient for practical applications.

Section 2 of this paper is devoted to summarising the theoretical formulations for the in-plane capacities of masonry walls provided by the current Italian building code [26,27]. 

Section 3 describes, in detail, the differences between the two macro-modelling approaches used in the parametric analyses, mainly in terms of input parameters and the modelling of materials, as well as the discretization method. Some experimental tests available in the literature are assumed as benchmarks for calibrating the two numerical models. As previously mentioned, this first calibration is aimed to check the possibility of simulating, reliably, the experimental conditions, not only by means of a refined FE model, but also by means of a simpler model (i.e., the DMEM), after a proper choice of input parameters, based on the comparison with experimental and numerical data.

Section 4 presents the results of several non-linear static analyses carried out on masonry walls by means of both FE and DME models and aiming to: (a) investigate the influence of the compressive and tensile strengths of masonry and of the fracture energy in compression and tensile of the masonry material on the whole behaviour of the masonry walls, in terms of both strength and ductility; (b) check the differences in the results provided by the FEM and DMEM as several parameter changes; (c) study the reliability of the code formulations and compare their predictions with the numerical results; (d) plan a suitable experimental program of shear-compression tests for masonry walls to check the effects of the most meaningful investigated parameters.

In the end, Section 5 deals with a useful discussion on the shape factor value used in the code formulations, based on the comparison between the numerical and theoretical results of the parametric analyses. 

## 2. Italian Code Formulations for the In-Plane Resistances of Masonry Walls

Several authors and codes have proposed different formulations to predict the in-plane resistances of masonry walls, for both flexural and shear failure modes. An accurate analysis of existing literature formulations is reported in Celano et al. [11], where the reliability of these walls has also been investigated by means of comparisons with a wide database of experimental results collected from the literature.

In order to highlight the most influencing parameters, and in view of the detailed discussion reported in [11], only the theoretical formulations provided by the current Italian building code [26,27] are introduced in the following. The effect of the influencing parameters is then investigated in Section 4 by means of several parametric analyses comparing the analytical results with the numerical models (FEM and DMEM).

### 2.1. In-Plane Strength Models for Irregular/Rubble Masonry Walls 

**Figure 1:**
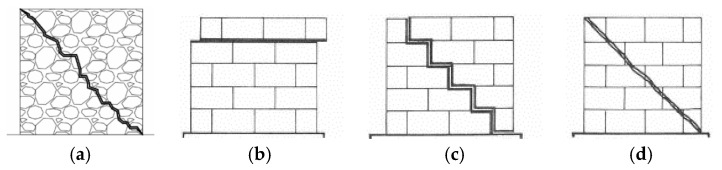
Different shear mechanisms in masonry walls: (**a**) diagonal shear failure (DS) in irregular masonry; (**b**) horizontal sliding shear failure (HSS) in regular masonry; (**c**) diagonal sliding shear failure (DSS) in regular masonry; (**d**) tensile diagonal cracking in the units (TDC) in regular masonry. Reprinted from Ref. [11].

Irregular/rubble masonry walls usually refers to walls made of irregular arrangements of units and mortar. Such irregularities influence the in-plane shear resistance of the wall, which can only occur for diagonal shear failure (Figure 1a), under the assumption of modelling the masonry as a macroscopic homogeneous material. This capacity is provided by the following formulation, based on the Turnšek and Čačovič model [32] and involving the tensile strength of masonry *f_t_*:

- diagonal shear failure (DS):

(1) $$V_{\mathrm{DS}}=B\cdot s\cdot \frac{f_{t}}{b}\cdot \sqrt{1+\frac{\sigma_{0}}{f_{t}}}$$

Additionally, the flexural strength is provided by the following equation:

- flexural failure (F):

(2) $$V_{F}=B^{2}s\cdot \frac{\sigma_{0}}{2H_{eff}}\cdot \left(1-\frac{\sigma_{0}}{0.85f_{c}}\right)$$

Both formulations depend on the geometrical parameters of the walls, i.e., the base *B*, the height *H*, and the thickness *s*, and on the vertical compressive stress σ_0_. Equation (1) also depends on the shape factor *b*, which should represent the maximum-to-average shear stress ratio along the middle cross-section of the wall. According to the Commentary to the Italian code [27], *b* is herein assumed equal to the in-plane slenderness of the wall *b* = *λ* = *H/B* but limited to the range 1.0–1.5. Equation (2) also depends on the compressive strength of masonry *f_c_*, and on the effective height *H_eff_*, assumed as the shear length and, thus, equal to 0.5 *H* in the case of double-fixed constraints.

The theoretical shear resistance is the minimum between the values provided by Equations (1) and (2).

### 2.2. In-Plane Strength Models for Regular Masonry Walls

Regular masonry walls are made of regular arrangements of units bonded with horizontal and vertical mortar joints. In addition to the flexural failure provided by Equation (2), two types of shear failure can occur, both related to sliding mechanisms: (a) sliding along aligned horizontal bed joints, namely horizontal sliding shear (HSS, Figure 1b), and (b) sliding along diagonal stepped cracks, namely diagonal sliding shear (DSS, Figure 1c). The following equations, both based on the well-known Mohr–Coulomb criterion, are provided by the Italian building code [26,27]:

- horizontal sliding shear failure (HSS):

(3) $$V_{\mathrm{HSS}}=B^{\prime }s\cdot \frac{f_{v0}+\mu \sigma_{0}}{\gamma_{d}}$$

- diagonal sliding shear failure (DSS):

(4) $$V_{\mathrm{DSS}}=\frac{Bs}{b}\cdot \left(f_{v0}^{\prime }+\mu^{\prime }\sigma_{0}\right)\leq V_{t,lim}$$

where:

(5) $$f_{v0}^{\prime }=\frac{f_{v0}}{1+\mu \varphi };\;\mu^{\prime }=\frac{\mu }{1+\mu \varphi };\;\varphi =2h_{b}/b_{b}$$

In Equation (3), *μ* is the friction coefficient and *f_v_*_0_ is the cohesion, defined as ‘local’ parameters, while in Equation (4) the so-called ‘global’ parameters *f*′*_v_*_0_ and *μ*′ are used in order to account for the interlocking between the units, which is expressed by means of the parameter *φ*, which depends on the height *h_b_* and the length *b_b_* of the units (Equation (5)). In both formulations, *σ*_0_ is the vertical compressive stress, while *B*′ is the reduced length of the end sections of the walls, corresponding to the compressed part of the section and intended to consider the cracks produced by the bending moment. Assuming a linear distribution of the compressive stresses and neglecting the tensile strength of the mortar, it is possible to calculate the reduced length *B*′ using the following formulation:

(6) $$B^{\prime }=3\cdot \left(\frac{B}{2}-e\right)$$

where *e* is the eccentricity of the vertical load [33].

The Italian code also suggests the following upper bound for the shear capacity related to the possible achievement of the tensile strength of the blocks *f_bt_* (TDC in Figure 1d):

- tensile diagonal cracking (TDC):

(7) $$V_{t,lim}=\frac{B\cdot s\cdot f_{bt}}{2.3\cdot b}\cdot \sqrt{1+\frac{\sigma_{0}}{f_{bt}}}$$

being *b* the same shape factor previously defined. Note that, lacking information on the tensile strength of the units, the Italian code suggests for *f_bt_* using 10% compressive strength of the unit, while Eurocode 6 [34] provides a lower percentage, i.e., about 3.2% compressive strength, which seems to be more realistic [11].

For flexural failure, Equation (2) is valid for regular masonry walls as well.

### 2.3. Considerations of In-Plane Strength Models for Regular and Irregular Masonry Walls

It is important to underline that the identification of the right cross-section of the wall is a fundamental point in the correct evaluation of its shear capacity, as discussed in [11]. In general, in the middle cross-section of a masonry wall, restrained in a double-fixed condition and tested according to a shear-compression test, the bending moment is zero, which means that the normal stresses are constant and equal to those applied on the top end section and only due to the vertical load. On the other hand, if the wall is assumed as a cantilever, the distribution of the normal stresses passes from a constant distribution at the top end section to a linear distribution at the fixed base end section, due to the linearity of the bending moment. 

Furthermore, the type of masonry plays a fundamental role in the choice of the right cross-section of the wall, due to the strength model assumed for calculating the shear capacity. For irregular masonry walls, indeed, as discussed in Section 2.1, Equation (1) [32], adopted by the Italian code, refers to the middle cross-section of the wall, since it has been shown that cracking phenomena start from the centre of the wall where the bending moment is negligible due to a double-fixed condition. On the contrary, for regular masonry walls, Section 2.2 has shown that the shear capacity is strongly influenced by the distribution of the normal stresses according to the Mohr–Coulomb criterion (Equations (3) and (4)). In this case, the middle cross-section of the wall is used as a reference section for the diagonal sliding shear failure (Equation (4)), while the base end section of the wall is assumed for the horizontal sliding shear failure (Equation (3)).

In this paper, the code formulation provided for the diagonal shear failure (DS) of irregular masonry walls will be adopted in favour of safety. In fact, as indicated by the Commentary to the Italian code [27] and as investigated in [11], Equation (1), i.e., the formulation suggested for the shear failure of irregular masonry walls, provides safe predictions for the shear failure of regular masonry walls as well. Moreover, the DS failure and the flexural one can be well-simulated by macro-modelling approaches, such as those used in this study, while the failure modes related to sliding phenomena can be properly simulated by means of micro-modelling approaches or other discrete macro-models [16], which are not examined in this paper. 

It is worth noting that Equation (1) has the advantage of also being usable to address the lack of information about the cohesion and friction angles required for regular masonry walls to apply Equations (3) and (4), and it has been verified that a proper calibration of the shape factor value *b* can assure the reliability of these formulations, even for regular masonry walls [11].

## 3. Description and Calibration of the Numerical Models

Section 2 demonstrates that the models predicting the in-plane shear resistances of masonry walls depend on a few mechanical properties of the masonry and geometrical data of the walls. However, when the in-plane behaviour is investigated by means of finite element models, several further mechanical parameters of masonry are required, and their choice may influence the results. Therefore, the parametric analyses presented in the following sections aim to both investigate the effect of the parameters considered by the code formulations and to assess the influence of the unconsidered ones. Moreover, because the reliability of non-linear FEMs requests a suitable assessment of the mechanical parameters of the masonry used—not always easily definable—as fracture energies, ultimate tensile strain and tensile strength, simpler models based on the macro-modelling strategy and adopting discrete macro-elements may be more convenient in terms of computational efforts, especially for the global analysis of whole buildings [35]. Therefore, coupled with a FE model implemented in the Diana FEA software [28], the parametric analyses are herein also carried out by means of a DMEM introduced in the 3DMacro software [29].

The two numerical models used in the parametric analyses reported in Section 4 are described in detail in the following.

### 3.1. Non-Linear FEM

A three-dimensional finite element model (FEM) has been implemented in the DIANA FEA software in order to model masonry walls under a constant compression stress and variable horizontal loads and to investigate, in detail, the in-plane behaviour. A macro-mechanical approach is used in the FEM to simulate the masonry, which is, thus, assumed as a continuous, homogeneous and isotropic material. The numerical analyses are performed by adopting the total strain crack model, which is based on the smeared crack model. In particular, a locally generalized crack is not modelled as a detachment between two surfaces (discrete crack model), but, rather, the material is considered always homogeneous and characterized by different mechanical properties after cracking. Moreover, in all the numerical analyses, the orientation of the cracks is assumed to be variable (rotating crack model).

**Figure 2:**
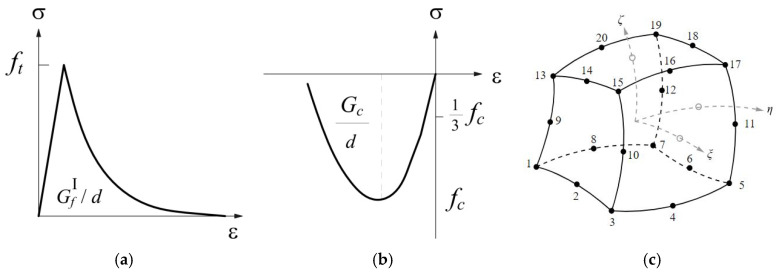
(**a**) Exponential tensile law, (**b**) parabolic compressive law, (**c**) CHX60, a type of solid element mesh.

The constitutive laws used for modelling the uniaxial behaviour of the homogenized material are plotted in Figure 2a,b for the tension and the compression behaviours, respectively. For tension, the linear behaviour until the tensile strength is followed by an exponential softening branch, while a parabolic law is assumed in compression, both before and after the peak strength. The mechanical properties required for the description of the masonry material are the Young’s modulus *E*, the Poisson ratio ν, the compressive strength of the masonry *f_c_*, the tensile strength *f_t_*, the fracture energies in compression *G_c_* and tension *G_t_*, which have been initially evaluated through the application of the following formulations provided in [36]:

(8) $$G_{c}=\left(2.8-0.1\cdot f_{c}\right)\cdot f_{c}$$

(9) $$G_{t}=0.025\cdot {\left(2\cdot f_{t}\right)}^{0.7}$$

A three-dimensional element, CX60, is adopted for the discretization of the continuum; it is a 20-node iso-parametric solid brick element based on a quadratic interpolation and Gauss integration (Figure 2c). The (secant) quasi-Newton method is used as an integration method, and normalized energy as a convergence criterion.

### 3.2. Non-Linear DMEM

**Figure 3:**
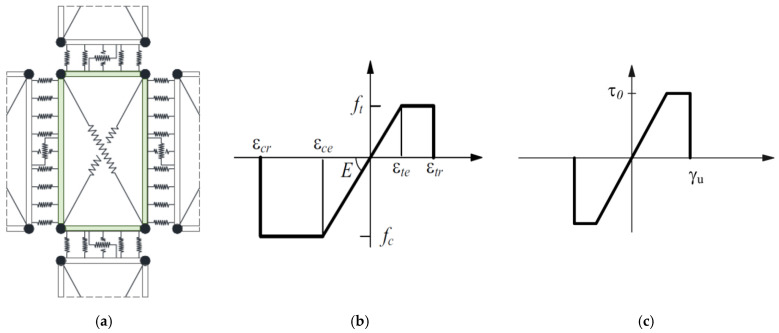
(**a**) 2D macro-element adopted in the 3D Macro software. Constitutive laws adopted in the macro-element model for: (**b**) tension/compression and (**c**) shear.

In the discrete macro-element model (DMEM) implemented in the 3DMacro software, the simulation of masonry elements is based on the definition of macro-elements. Each macro-element is made of four hinges connecting four rigid one-dimensional elements and two diagonal non-linear springs (Figure 3a) [31,37]. This approach catches the main in-plane failure mechanisms of a masonry panel (flexural, diagonal shear and sliding shear failures) by means of a reduced number of parameters. The activation of a flexural failure is controlled by the orthogonal springs along the interface elements, while the diagonal shear behaviour is governed by the non-linear diagonal springs and the sliding shear failure by additional non-linear longitudinal springs along the interface elements (Figure 3a). Non-linear springs are used to take into account the mechanical properties of masonry, and the spacing among the springs at the interface elements is calibrated equal to 20 mm.

Since different constitutive laws of masonry are used within the FEM and DMEM, it is necessary to calibrate some parameters of the DMEM in order to compare the results correctly. The elastic-perfectly plastic law for masonry used in the DMEM (Figure 3b) requires the definition of the ductility of masonry both in terms of compression β*_c_* and tension β*_t_* and each is expressed as the ratio of the related ultimate strain (ε*_cr_* or ε*_tr_*) to the limit elastic one (ε*_ce_* or ε*_te_*). Specifically, the plastic phase area of the constitutive law plotted in Figure 3b is matched to the fracture energy used in the FEM (Figure 2a,b), adopting the following correlations:

(10) $$\varepsilon_{cr}-\varepsilon_{ce}=\left(\frac{G_{c}}{d}\right)/f_{c}\;\mathrm{for}\;\mathrm{compression}$$

(11) $$\varepsilon_{tr}-\varepsilon_{te}=\left(\frac{G_{t}}{d}\right)/f_{t}\;\mathrm{for}\;\mathrm{tension}$$

where *d* is the diagonal of the mesh size adopted in the FEM, and *f_c_* and *f_t_* have the same meaning and value adopted in the FEM.

The shear behaviour of masonry is defined by the elastic-plastic law plotted in Figure 3, where the stiffness is defined through the shear modulus *G*, based on the values of *E* and ν (Poisson’s ratio), the pure shear strength τ_0_ in absence of compression stress is calculated by dividing the masonry tensile strength *f_t_* to 1.5, and the shear strain capacity *γ_u_* is fixed equal to 0.5% of the height of the macro-element in compliance with the Italian NTC 2018 [26]. For calculating the shear resistance, the Turnšek and Čačovič criterion [32] is adopted that corresponds, thus, to assume a diagonal shear (DS) failure.

### 3.3. Calibration of the Numerical Models against Experimental Case Studies

**Figure 4:**
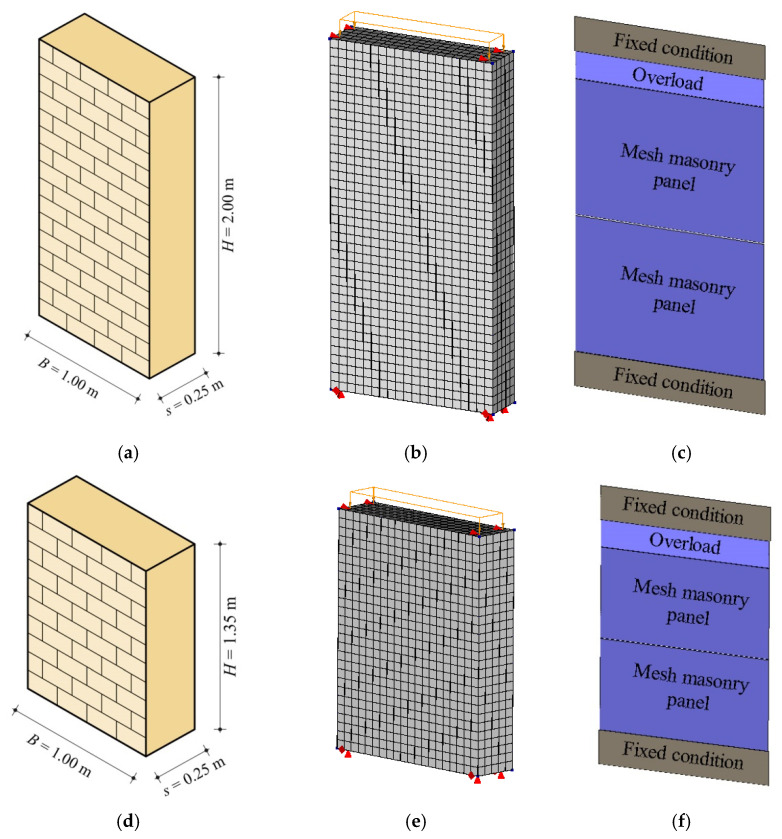
Slender wall [22]: (**a**) geometry; (**b**) FE model; (**c**) DME model. Squat wall [22]: (**d**) geometry; (**e**) FE model; (**f**) DME model.

Before carrying out parametric analyses, the reliability of both numerical models has been checked by means of comparisons with the experimental tests of Anthoine et al. [22], assumed as a benchmark. The experimental tests were executed on two masonry walls made of clay bricks according to a ‘regular’ texture, characterized by two slenderness ratios λ = *H*/*B* = 2 and 1.35 (Figure 4a,d), with heights *H* = 2000 mm and *H* = 1350 mm, respectively, base *B* = 1000 mm, and thickness *s* = 250 mm. The computational models corresponding to the two walls are illustrated in Figure 4b,e for FEM and in Figure 4c,d, for DMEM. In our experimental set-up, the tested masonry walls were restrained according to the double-fixed boundary condition.

The two slenderness ratios were originally chosen by the authors [22] in order to catch two different failure mechanisms in the walls under the same vertical compression load, i.e., a constant pre-compression stress σ_0_ = 0.6 MPa. The slender wall failed, indeed, for flexure (F), while the squat wall attained a diagonal sliding shear (DSS) failure.

The values of the compressive and tensile strength of masonry provided by the authors [22] were *f_c_* = 6.20 MPa and *f_t_* = 0.25 MPa, respectively. Lacking detailed experimental information on the Young’s modulus *E* of masonry, this parameter has been assumed equal to 1700 MPa, based on the best fitting between the experimental results and the numerical load-displacement curves obtained by the previously described modelling approaches.

For the FEM, the fracture energies in compression and tension *G_c_* and *G_t_* are evaluated according to Equations (8) and (9), respectively, and are *G_c_* = 10 N/mm and *G_t_* = 0.012 N/mm, based on the values of *f_c_* and *f_t_*. The Poisson’s ratio is assumed ν = 0.2. An optimization process of the mesh has been carried out and the best mesh size, in terms of reliability of result, and computational effort is identified in 50 mm × 50 mm, whose diagonal *d* is used in Equations (10) and (11).

For the DMEM, further parameters need to be estimated through the equivalence described above; in particular, the values of ductility in compression and tension are β*_c_* = 4.6 and β*_t_* = 3.3, while the normal and the shear elastic moduli of masonry are *E* = 1943 MPa and *G* = 816 MPa, respectively. The mesh size adopted in the DMEM is 1000 mm × 1000 mm, which, again, represent a good balance between the reliability of results and the computational effort.

The values of all the mechanical parameters of masonry adopted in the FEM and DMEM are listed in Table 1. In both software, the numerical analyses are performed under displacement control, with the aim of capturing the post-peak behaviour of the wall, as well.

**Figure 5:**
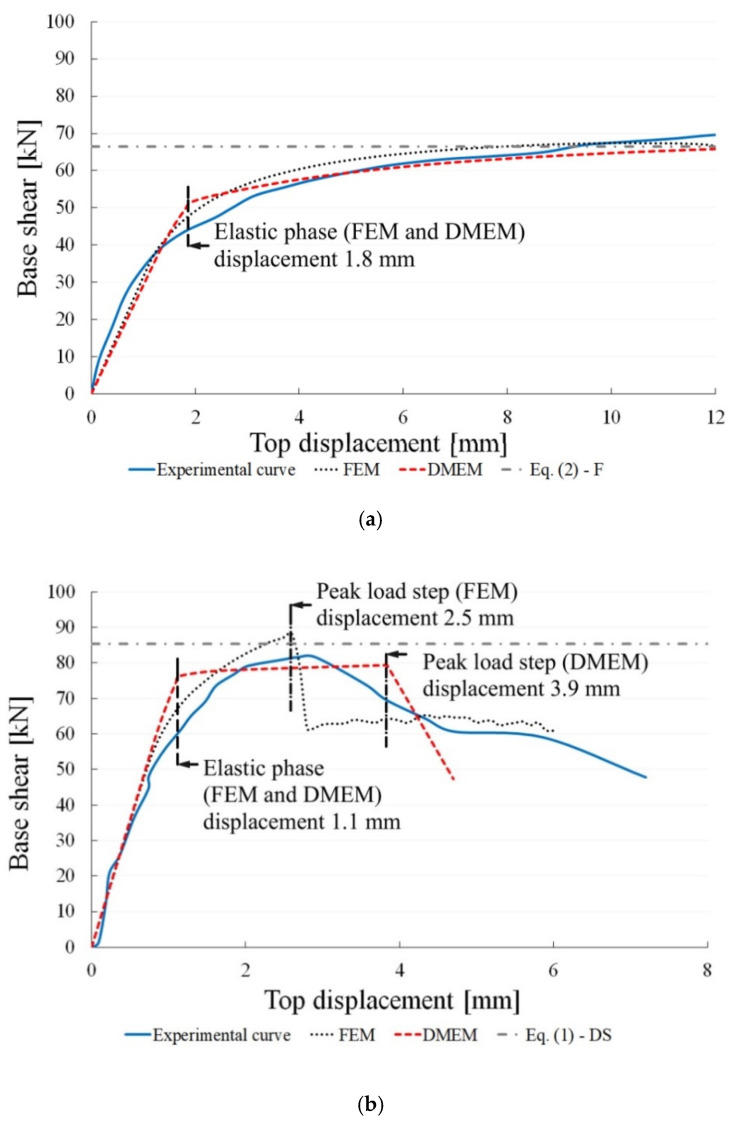
Experimental vs. numerical curves given by the FEM and DMEM for: (**a**) slender and (**b**) squat walls.

To validate the calibration of the two models, the numerical results are compared in Figure 5 with the experimental ones presented in [22], with reference to both the slender and the squat walls experimentally tested. In Figure 5, the analytical predictions given by Equations (1) and (2) are reported as well. It is worth highlighting that, although the experimentally tested walls are made of regular masonry, for their shear failures, only the formulation for irregular masonry walls is herein adopted for the previously described reasons: (1) Equation (1) can provide safe results for regular masonry walls as well; (2) the local or global values of cohesion and friction angle are not provided; (3) in the FEM, the homogenized approach used for masonry is not able to simulate sliding mechanisms along the mortar joints, typical of regular masonry walls, but, rather, only the diagonal shear failure analytically represented by Equation (1).

In Table 2, the values of the analytical shear resistance provided by Equations (1) and (2) are listed together with the numerical predictions and the experimental results. It can be observed that, for the slender wall, the flexural capacity given by Equation (2) is lower than the shear one provided by Equation (1), confirming, thus, the experimental and numerical failure mode due to bending; moreover, Equation (2) provides a capacity only 5% lower than the experimental one and in agreement with the numerical ones (difference of about 3%). On the other hand, for the squat wall, the shear capacity given by Equation (1) is lower than the flexural one, confirming also in this case the experimentally observed failure mode, i.e., the shear one. 

Although the experimental failure was due to the horizontal sliding shear (HSS), the analytical diagonal shear resistance (DS) given by Equation (1) overestimates the experimental one by only 5%, confirming the good reliability of Equation (1) also for regular masonry walls. Equation (1) is also in quite good agreement with both numerical predictions, with differences ranging between −3% for the FEM and +7% for the DMEM. Such agreement is justified by the fact that both the numerical models adopt the same homogenized approach used in the strength model represented by Equation (1), which is based on the tensile shear failure of masonry. In conclusion, the comparisons of the numerical results with the experimental ones and the analytical ones do confirm the soundness of the two modelling strategies.

The shear–displacement curves plotted in Figure 5 show, for the slender wall (Figure 5a), a good agreement between the experimental and numerical curves provided by both models. There is only a slight difference in terms of initial stiffness, which is lower than that observed in the experimental curve for both numerical models.

For the squat wall (Figure 5b), the agreement between the numerical and experimental curves is very good in terms of initial stiffness, while the numerical post-peak phases are quite different from the experimental one and are different from each other. The discrepancy between the two numerical models is due to the different constitutive laws used for modelling the masonry wall and the different strategies for modelling the shear behaviour. The FEM is able to better catch the experimental peak load, even if the softening behaviour is characterized by a brittle reduction followed by a quasi-horizontal branch, while the experimental one shows a less steep behaviour after the peak. Moreover, the experimental curve shows a larger ductility in comparison with both numerical curves. The differences between the numerical and the experimental curves can also be identified in the homogenized approach used for masonry that, as previously evidenced, is not able to simulate the sliding mechanism along the mortar joints, which is typical of regular masonry and, indeed, also occurred in the examined experimental tests. This might be also a reason of the higher ductility evidenced in the experimental curve since the sliding mechanisms provide a less brittle failure in comparison with the diagonal shear one.

However, the good approximations attained by both the numerical results evidence that they may provide reliable predictions for regular masonry walls, even if they are based on failure mechanisms typical of irregular masonry walls. This allows the generalization of the results provided by the FEM and DMEM to any type of masonry and confirms indicating [27] the safe use of Equation (1) for any masonry type.

**Figure 6:**
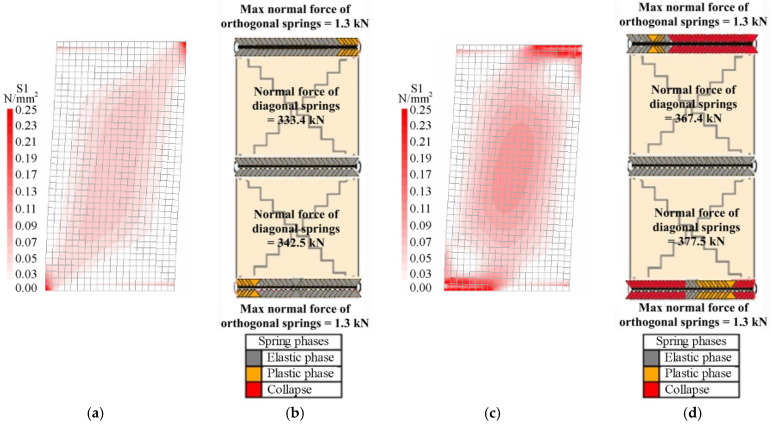
Distributions of the principal stresses and normal forces into the slender masonry panel at the end of the elastic field: (**a**) FEM, (**b**) DMEM, and at the peak load step: (**c**) FEM, (**d**) DMEM.

**Figure 7:**
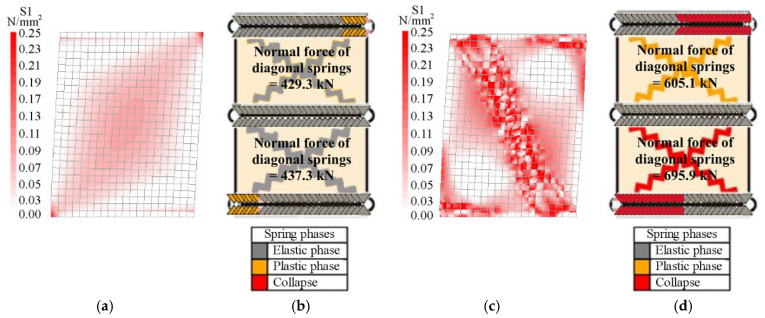
Distributions of the principal stresses and normal forces into the squat masonry panel at the end of the elastic field: (**a**) FEM, (**b**) DMEM, and at the peak load step: (**c**) FEM, (**d**) DMEM.

Figure 6 and Figure 7 show the distributions of the principal tension stresses for the FEM, called S1, and the normal forces in the springs for the DMEM, calculated for both models, either at the end of the elastic linear branch and at the peak load step (see thresholds indicated in Figure 5). In particular, the end of the elastic phase corresponds to the displacement of 1.1 mm for the squat wall and 1.8 mm for the slender wall. The displacement of the peak load step, related to the squat masonry panel, is assumed equal to 2.5 mm and 3.9 for the FE and DME models, respectively. On the other hand, the peak load step for the slender masonry panel corresponds for both models at the displacement of 12 mm where the analyses have been interrupted as in the experimental tests. 

Figure 6a and Figure 7a respectively show that, according to the FEM, the tension stress distributions in the two walls are initially very similar because masonry behaves as elastic in all regions of the walls, while they are significantly different when the maximum loads are reached. In fact, at the peak load step, the stresses are mainly concentrated at the two end sections in the slender panel (Figure 6c) due to the flexural failure (F) and along the compressed diagonal in the squat panel (Figure 7c) due to the diagonal shear failure (DS), where the cracking phenomena occur. Note that the value of 0.25 MPa in the legends for the FEM corresponds to the tensile strength of masonry.

On the other hand, the results of the DMEM are not directly comparable with the those of the FEM, because of the different numerical approaches previously discussed. However, some useful considerations can easily be derived in terms of the normal forces acting in the non-linear springs of the DMEM.

Bearing in mind that the activation of the flexural failure for the DMEM is controlled by the orthogonal springs along the interface elements, Figure 6b,d and Figure 7b,d show the normal forces acting in the springs at the constrained sections of the slender and squat masonry panel, in the elastic field and at the peak load step, respectively. As expected, since the slender wall (Figure 6b,d) failed in flexure, most of these springs are collapsed (red colour) at the peak load step, while at the end of the elastic field almost all of them are still in the elastic phase (grey colour). Moreover, it can be noted that the diagonal springs remain in the elastic phase. Conversely, for the squat masonry, Figure 7b with Figure 7d evidence a clear increasing of the normal forces in the two diagonal springs in comparison with the slender wall (about +50%), because of the shear failure.

The representation of the internal stress distributions at the two load steps allows understanding the evolution of the cracking phenomena inside the panels. For the slender wall, the cracks start to develop at the end sections of the panel and continue increasing mainly there during the loading process, while, for the squat wall, the cracking phenomena start at the two edges of the diagonal in tension and then continue in the centre of the panel due to a variation in the distribution of the stresses. This phenomenon can be explained by considering that a strut and tie mechanism tends to develop inside the squat panel, with a concentration of the maximum tensile stresses along the compressed diagonal (Figure 7c).

Another important aspect that can be derived from Figure 6 and Figure 7 is the effect of the constraints. In fact, at both the restrained ends of the wall, the supports tend to absorb part of the tension that arises during the application of the horizontal load. As the load increases, this restraint effect and the consequent cracking tend to reduce the compressed zones of the cross-sections at both the ends of the wall due to the increase of the related bending moments.

**Figure 8:**
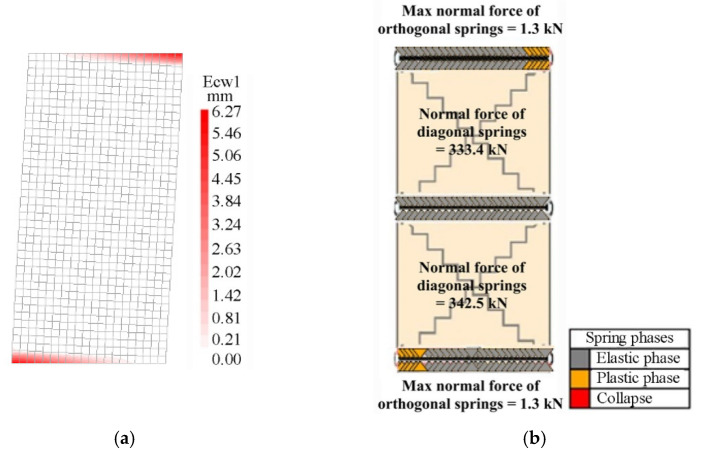
Comparison between the crack patterns in the slender masonry panel at the peak load step: (**a**) FEM, (**b**) DMEM.

**Figure 9:**
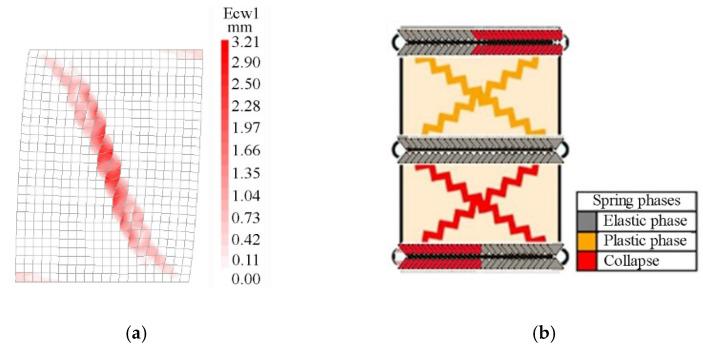
Comparison between the crack patterns in the squat masonry panel at the peak load step: (**a**) FEM, (**b**) DMEM.

Figure 8 and Figure 9 show the comparison between the numerical crack patterns (called Ecw1 in the FEM) at the peak load, for the slender and the squat walls, respectively. The slender wall exhibits a flexural failure, with the typical distribution of the cracks at the two end sections of the wall restrained in a double-fixed condition, while the squat wall shows a shear failure. The experimental crack patterns described by Anthoine et al. [22] are similar to the numerical ones but developed in two directions, since a quasi-static cyclic loading condition was applied.

## 4. Parametric Analyses and Comparison with Theoretical Formulations

**Figure 10:**
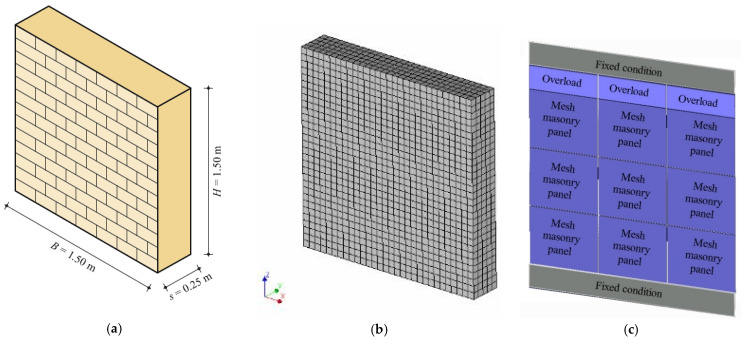
(**a**) Geometry of the masonry panel used for the parametric analyses and computational models: (**b**) FEM and (**c**) DMEM.

After the reliability of the FEM and DMEM has been checked by means of comparisons with experimental results and code formulations, the two approaches are herein used for carrying out sensitivity analyses on squat masonry walls subjected to the shear-compression loading configuration changing several mechanical parameters. Squat walls are only considered herein, to focus attention mostly on shear failures and, thus, their geometrical dimensions, i.e., *H* = 1500 mm, *B* = 1500 mm, *s* = 250 mm, slenderness λ = *H*/*B* = 1, are kept fixed in all the analyses (Figure 10a). As for the case study previously described, the walls are supposed fixed at the two ends in order to reproduce the boundary condition of masonry piers. Figure 10b,c show the mesh discretization adopted in the two models, FEM and DMEM respectively, with the mesh size previously defined. 

### 4.1. Definition of Ranges of Variability for the Mechanical Parameters

The mechanical properties considered in the parametric analyses are gathered in four groups in Table 3, each of which referred to a single variable parameter assumed as a main one. Since the vertical compression significantly influences the in-plane response of masonry walls under shear actions, the values of 0.3 MPa and 0.6 MPa are chosen as representative of realistic compressive stress conditions in existing masonry buildings.

The four groups of analysis are:

- Group A (Table 3): refers to the choice of four types of masonry characterized by different values of the compressive strength *f_c_*, assumed as main parameter and variable in the range 1.5–6.0 MPa. All the remaining parameters vary according to *f_c_*, as follows: *f_t_* is assumed as 5% of *f_c_*, τ_0_ is equal to *f_t_*/1.5, *G_c_* and *G_t_* are obtained by applying Equations (8) and (9) based on the values of *f_t_* and *f_c_*;
- Group B (Table 4): refers to the variation of the tensile strength *f_t_*, which is assumed as a main parameter and variable in the range 0.08–0.45 MPa; the related parameters (i.e., fracture energy in tension and ultimate strain in tension) are varied consequently for two levels of σ_0_ and three values of *f_c_*;
- Group C (Table 5): refers to the variation of the fracture energy in compression *G_c_*, which changes in the range 2–10.6 N/mm and determines the consequent variation of the ultimate strains in compression ε*_cr_* for two levels of σ_0_ and two values of *f_c_*;
- Group D (Table 6): refers to the variation of the fracture energy in tension *G_t_*, which changes in the range 0.003–0.05 N/mm and determines the related variation of the ultimate strains in tension ε*_tr_* for two levels of σ_0_ and two values of *f_c_*.

The choice of the ranges of variation for the tensile and compressive strengths as well as for the Young’s modulus is based on the nominal values provided by the Commentary to the Italian code [27] for existing masonry buildings. Note that the cases of Group A highlighted in bold are also contained in the other groups and, thus, their results are reported more than once.

In the following, four subsections are presented, one for each group of analysis. In each subsection, the numerical shear resistances provided by the FEM and DMEM and the corresponding failure modes predicted by each model are compared between them and with the theoretical results given by Equations (1) and (2), i.e., *V*_DS_ and *V*_F_.

In order to investigate the effect of the variation of the main parameters, for each numerical model, the percentage variation, Δ*V*, is reported with respect to the case with the lowest value of the main varied parameter in each subgroup.

Additionally, to check the difference in the results provided by the two numerical models, for each examined case, the percentage variation of the predicted capacities is evaluated as follows:

(12) $$\Delta V_{num}=\frac{V_{\mathrm{DMEM}}-V_{\mathrm{FEM}}}{V_{\mathrm{FEM}}}$$

where *V*_FEM_ and *V*_DMEM_ are the numerical resistances provided by the FEM and DMEM, respectively.

Finally, in order to compare the results of the two numerical models with the theoretical predictions, the percentage difference of the theoretical capacity *V_th_*, corresponding to the failure mode predicted by the numerical models is evaluated as follows:

(13) $$\Delta V_{th}=\frac{V_{th}-V_{NUM}}{V_{NUM}}$$

where *V_NUM_* is *V*_FEM_ or *V*_DMEM_ in turn and *V_th_* is *V*_DS_ or *V*_F_, depending on the numerical failure mode.

**Figure 11:**
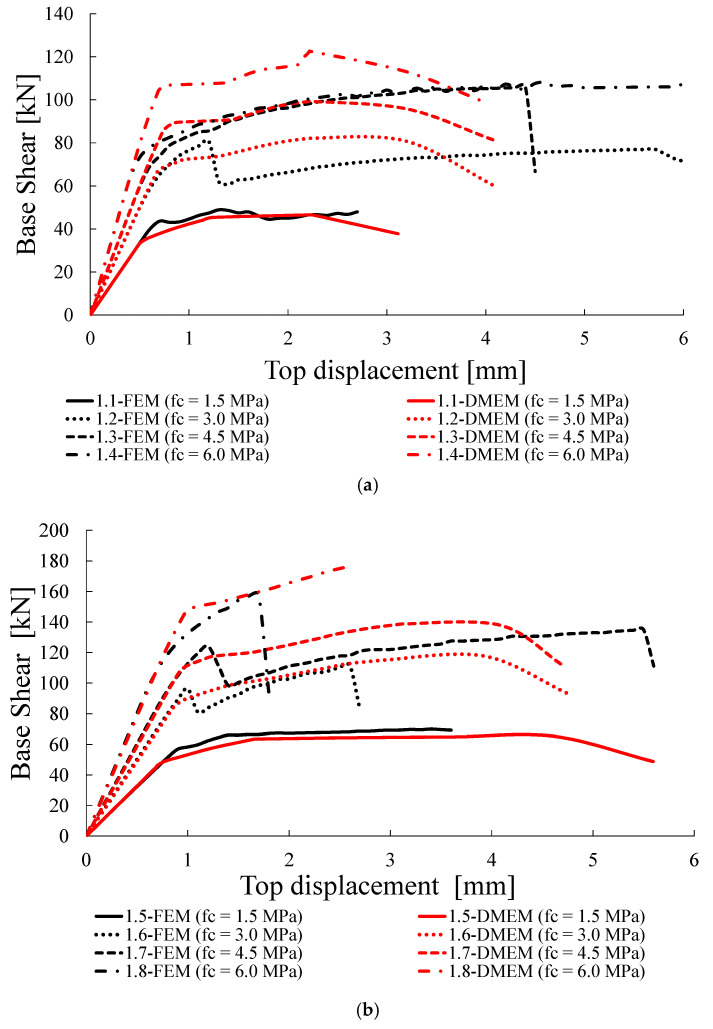
Capacity curves for Group A: (**a**) σ_0_ = 0.3 MPa, (**b**) σ_0_ = 0.6 MPa.

**Figure 12:**
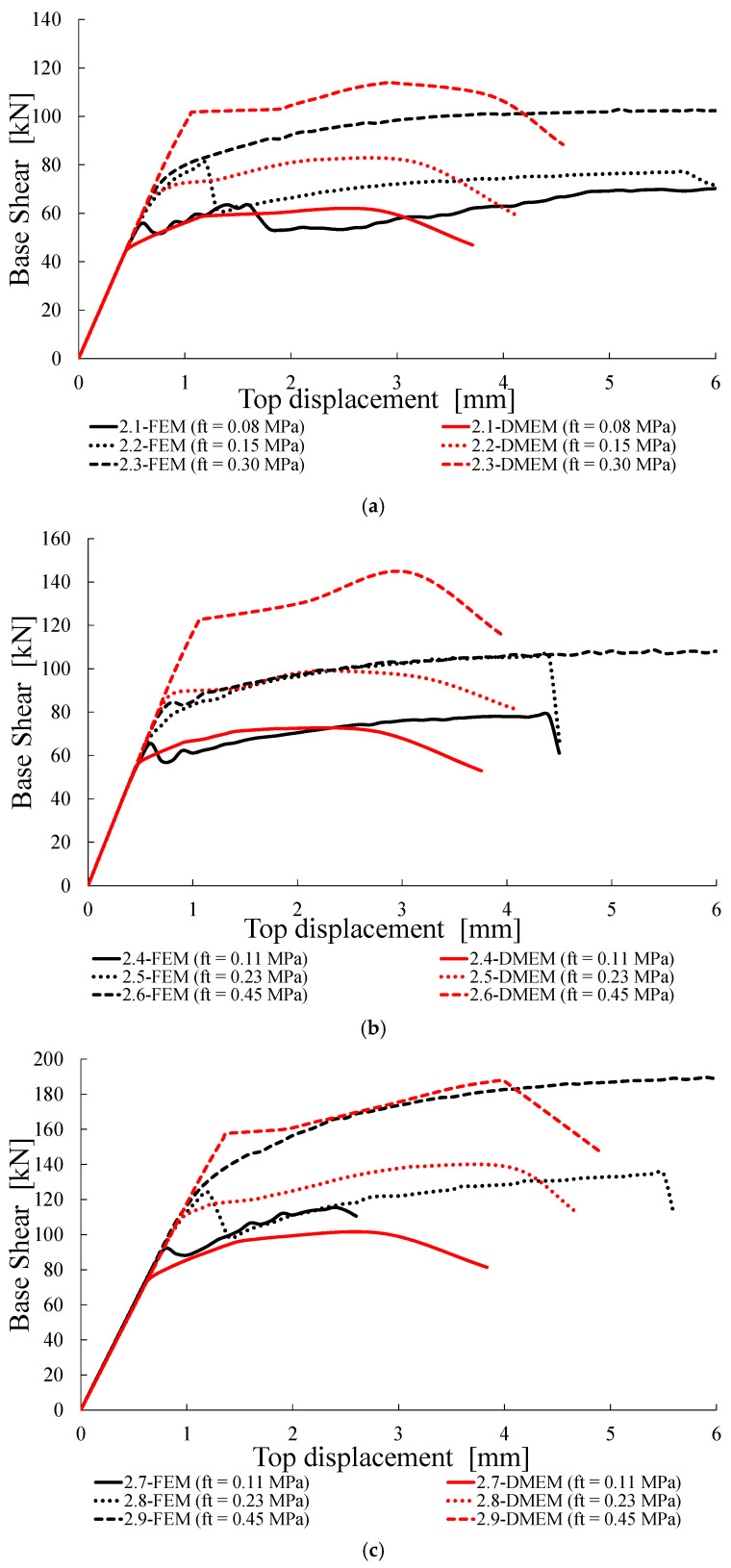

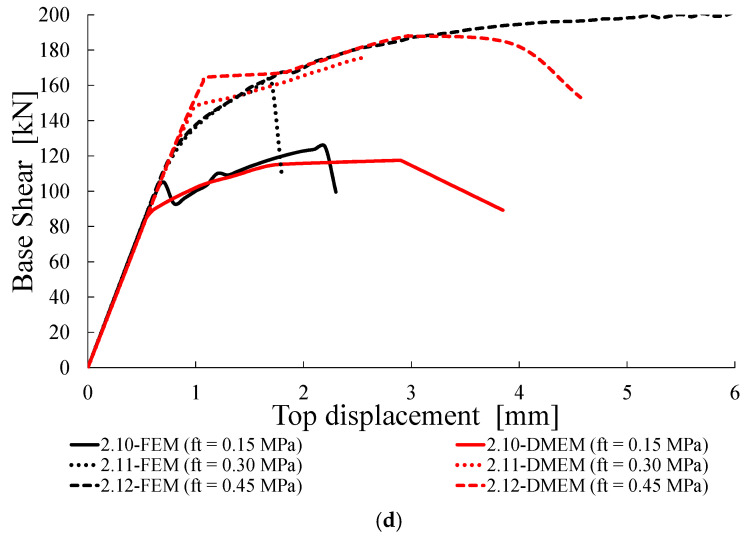
Capacity curves for Group B: (**a**) σ_0_ = 0.3 MPa and *f_c_* = 3.0 MPa, (**b**) σ_0_ = 0.3 MPa and *f_c_* = 4.5 MPa, (**c**) σ_0_ = 0.6 MPa and *f_c_* = 4.5 MPa, (**d**) σ_0_ = 0.6 MPa and *f_c_* = 6.0 MPa.

**Figure 13:**
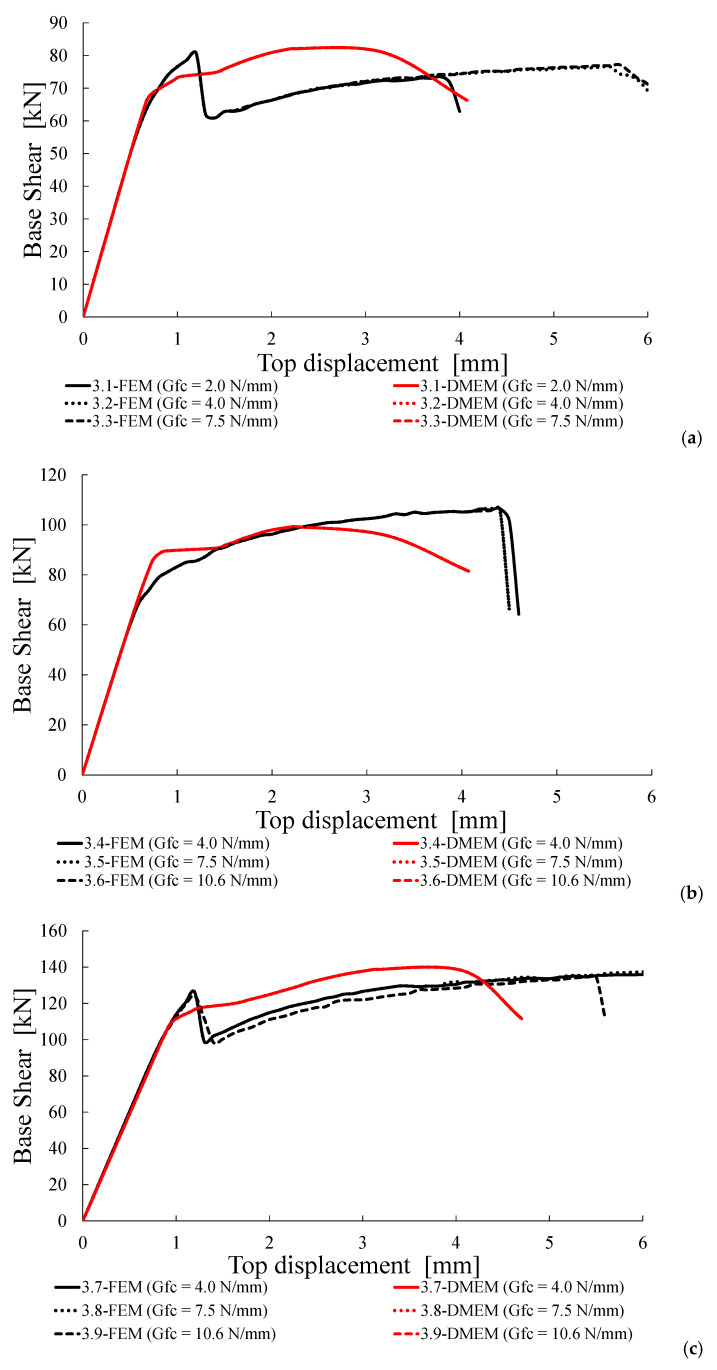
Capacity curves for Group C: (**a**) σ_0_ = 0.3 MPa and *f_c_* = 3.0 MPa, (**b**) σ_0_ = 0.3 MPa and *f_c_* = 4.5 MPa, (**c**) σ_0_ = 0.6 MPa and *f_c_* = 4.5 MPa.

**Figure 14:**
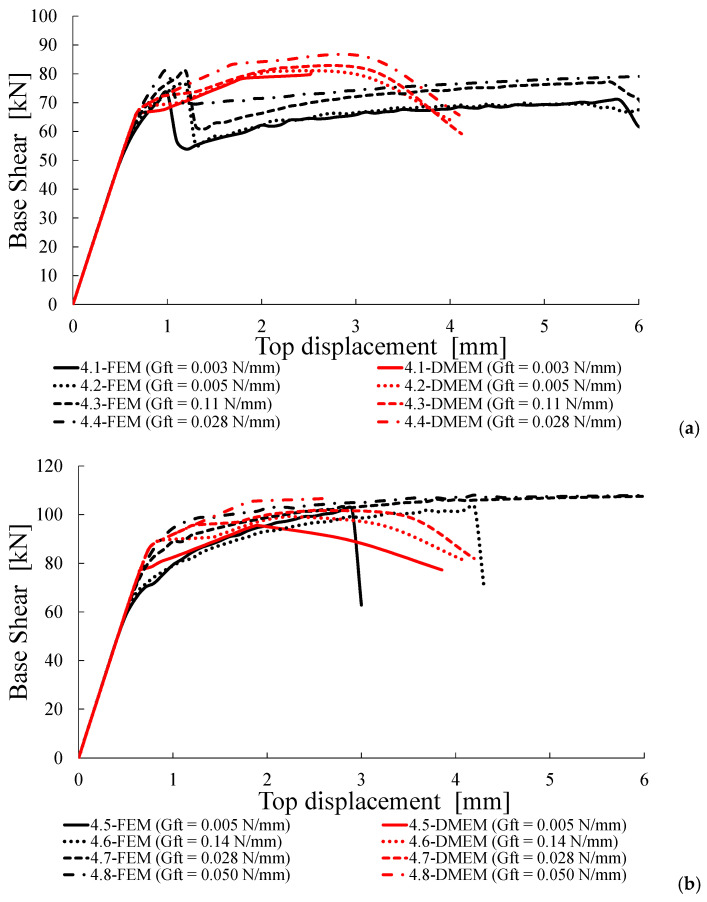

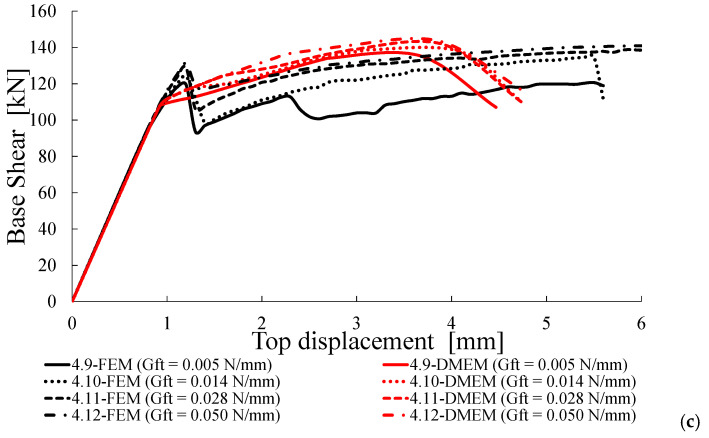
Capacity curves for Group D: (**a**) σ_0_ = 0.3 MPa and *f_c_* = 3.0 MPa, (**b**) σ_0_ = 0.3 MPa and *f_c_* = 4.5 MPa, (**c**) σ_0_ = 0.6 MPa and *f_c_* = 4.5 MPa.

Successively, the numerical load-displacement curves provided by the FEM and DMEM are plotted in Figure 11, Figure 12, Figure 13 and Figure 14.

The capacity curves obtained with numerical models are limited in all cases to the displacement of 6 mm, which, for the examined geometry, corresponds to a drift of 0.4%, in order to make the graphs clear and comparable, if any failure can be predicted before with a capacity decreasing higher than 30%. Note that the maximum drift provided by in case of shear failure is 0.5%. Nevertheless, it is worth noting that in both software programs, the pushover curves are mostly interrupted earlier, due to numerical convergence issues.

Also, it is important to highlight that the crack patterns referred to the single cases described in the following are similar to those already reported and discussed in Section 3, i.e., those for the flexural failure (F) in Figure 8 and those for the diagonal shear failure (DS) in Figure 9. For this reason, it has been decided not to report the stress/deformation distributions for the numerical analyses presented in the following sections.

### 4.2. Group A: Compression Strength f_c_ Variable

A first comparison can be obtained by varying the compressive strength *f_c_*, as reported in Table 7. The two numerical models give the same failure modes and very similar results in terms of loads, with the differences Δ*V_num_* varying from −9.0% to +13.4%.

Most walls fail in shear and only two cases, 1.3 and 1.4, are characterized by the highest compressive strengths and the lowest level of pre-compression, fail in flexure, in perfect agreement with the theoretical predictions, i.e., Equation (2). In most cases of shear failure, Equation (1) tends to overestimate the capacities provided by the numerical models, with a slightly higher overestimation with reference to the FEM (Δ*V_th_* varying in the range 13–21%). Only for the two cases, i.e., 1.1 and 1.5, characterized by the lowest compressive and tensile strengths of masonry, Equation (1) provides slightly lower predictions than the numerical ones.

As expected, increasing the strength in compression the wall capacity also increases, but less than proportionally. For instance, according to the FEM, the values of Δ*V* listed in Table 7 shows that the capacity of the walls increases from +65% to +121% for σ_0_ = 0.3 MPa and from +55% to +127% for σ_0_ = 0.6 MPa, while the compressive strength is between two and four times higher than the minimum value adopted in this set of analysis, i.e., 1.5 MPa. A similar trend of Δ*V* is detected for the DMEM as well.

Again, according to the FEM, for the same values of compressive strength, the increase of σ_0_ from 0.3 to 0.6 MPa determines the increases of the wall resistances by +47%, +38% + 27% and +51% for *f_c_* = 1.5 MPa, 3.0 MPa, 4.5 MPa and 6 MPa, respectively. This means that the compression stresses acting in the walls may significantly improve the shear strength.

Figure 11a,b show the capacity curves provided by the two software for the case σ_0_ = 0.3 MPa and 0.6 MPa, respectively. In addition to the good agreement, in terms of capacity, commented upon above, there is a very good correspondence along the elastic branches in terms of stiffness, while some differences occur when the non-linear behaviour is attained, especially for the higher compressive strengths. For the cases with *f_c_* = 6.0 MPa (1.4 and 1.8), there is, indeed, the maximum difference in terms of resistance provided by the two models (Δ*V_num_*= 13.4% and 8.3%, respectively).

It can be observed that for the cases 1.6 and 1.7 in the FEM, the shear suddenly decreases at a displacement of about 1 mm, due to the development of a shear crack. However, being a reduction lower than 30%, it does not prevent the curve from running.

### 4.3. Group B: Tensile Strength f_t_ Variable

The effects of the variation of the tensile strength *f_t_* on the in-plane resistance and the non-linear behaviour of the wall are reported in Table 8. Firstly, it can be noted that the failure modes predicted by the FEM and DMEM are always the same and, in most cases, also coincide with the theoretical ones. The only exception is the case 2.5, for which the theoretical flexural strength, given by Equation (2), is lower than the shear one, given by Equation (1), contrarily to what predicted by the numerical models.

In the cases of flexural failure, the values of Δ*V_th_* for the FEM predictions are lower than −5%, while, for the DMEM, the differences are more variable (Δ*V_th_* varies from −28% to +6%).

On the other hand, in the cases of shear failure, Equation (1) provides more scattered values of capacity in comparison with the FEM predictions (both higher and lower values are, indeed, provided with Δ*V_th_* variable from −13% to +21%). Conversely, Equation (1) gives differences with the DMEM predictions varying from +3% to +19%, being overestimations in all the cases. It is interesting to note that this overestimation of Equation (1) is higher for the higher values of *f_t_*, meaning that its dependence on the tensile strength is not exactly the same as that provided by the DMEM.

Actually, in both numerical models the shear capacity increases with the tensile strength according to a less than proportional trend. With reference to the results of the FEM, indeed, in the case of σ_0_ = 0.3 MPa, the wall resistance increases by 42% and 39% for *f_c_* = 3.0 MPa and 4.5 MPa, respectively, when *f_t_* increases about 4 times. For σ_0_ = 0.6 MPa, the wall resistance increasing is further higher, i.e., 64% and 61% for *f_c_* = 4.5 MPa and 6.0 MPa, respectively, when *f_t_* increases about four-fold. It can also be observed that the variations Δ*V* with *f_t_* are less significant than those derived with *f_c_* in Table 7.

Finally, it can be noted that the two numerical models give similar results, with differences Δ*V_num_* that vary from −15% to +11%. Only for the case 2.6 is there a relevant discrepancy of the results (+33% of DMEM vs. FEM), probably due to an anomalous peak in the DMEM capacity curve for a displacement of about 3 mm.

Figure 12 shows all the shear-displacement curves obtained for the cases listed in Table 8. It can be observed that, in most cases and for both models, the increasing of *f_t_* also increases the ultimate displacement of the wall and makes the capacity curves less stepped. While the elastic branches are coincident for all the cases, some differences in the capacity curves provided by the two models can be detected.

### 4.4. Group C: Fracture Energy in Compression G_c_ Variable

In this group of analysis, the fracture energy in compression, *G_c_*, is assumed to be variable with respect to the values calculated by means of Equation (8) for *f_c_* equal to 3.0 and 4.5 MPa. In Group C, indeed, for *f_c_* = 3 MPa it is assumed σ_0_ = 0.3 MPa and *G_c_* variable (i.e., lower values respect to those provided by Equation (8)), while for *f_c_* = 4.5 MPa it is assumed σ_0_ = 0.3 MPa and 0.6 MPa with and *G_c_* variable with the same trend.

As expected, and shown in Table 9, the fracture energy in compression impacts only for the FEM, though small, but has no influence for the DMEM and clearly for the theoretical formulations (Equations (1) and (2)), since it is not a parameter included in these strength models. This is a confirmation that *G_c_* is not relevant when the shear failures are related to the achievement of the tensile strength of masonry along diagonal cracks. The failure loads and modes of the examined cases only depend on the compressive and tensile strengths, as well as on the pre-compression level assumed in the analysis.

For both numerical models, the shear failure mode is always attained with very similar results (i.e., Δ*V_num_* varies from −7% to +3%). Equation (1) always overestimates the numerical shear capacities with values of Δ*V_th_* in the range of 16–21% when the theoretical shear capacity is lower than the flexural one. When *V*_DS_ is greater than *V*_F_, the overestimation of the numerical values provided by Equation (1) is further higher (between +21 and +30%).

Figure 13 shows the capacity curves provided by the two software for the case σ_0_ = 0.3 MPa (Figure 13a,b) and 0.6 MPa (Figure 13c). For the DMEM there is no influence of *G_c_* on the capacity curve, while for the FEM it slightly affects the ultimate displacement. Both programs provide the same trends in the elastic branches, while some differences occur when the non-linear behaviour is attained.

### 4.5. Group D: Fracture Energy in Tension G_t_ Variable

The fourth group of analyses evaluates the effect of the variation of the fracture energy in tension, *G_t_*, by assuming values different from that provided by Equation (9) for *f_t_* equal to 0.15 and 0.23 MPa. In Group D, for *f_t_* = 0.15 MPa, it is assumed σ_0_ = 0.3 MPa and *G_t_* variable, while, for *f_t_* = 0.23 MPa, it is assumed σ_0_ = 0.3 MPa and 0.6 MPa with *G_t_* variable. In all cases, lower and higher values of *G_t_* with respect to those provided by Equation (9) are considered.

Table 10 shows that for both models, the variation of *G_t_* does not significantly modify the shear capacity of the walls. In fact, the capacity increases until 12% according to the DMEM for the cases with σ_0_ = 0.3 MPa and *f_c_* = 3.0 MPa and until 15% according to the FEM for the cases with σ_0_ = 0.6 MPa and *f_c_* = 4.5 MPa. In most cases, the shear resistances provided by the two models are in good agreement, with a maximum variation Δ*V_num_* equal to about 9%. The failure modes predicted by the two approaches are always the same, i.e., the shear failure in all cases with exception of case 4.8 characterized by a flexural failure. The shear failure is also predicted by Equations (1) and (2) in most cases, with exception of cases from 4.5 to 4.8 where Equation (2) gives lower values of strength and, thus, a flexural failure is predicted.

In the cases of shear failure provided by both numerical models and Equations (1) and (2), Equation (1) overestimates the numerical results; the differences Δ*V_th_* between the FEM results and Equation (1) range, indeed, from about +11% to +33%, with the lower values attained for the cases of higher pre-compression levels and higher *f_c_* (i.e., the last subgroup in Table 10). For the DMEM, the difference on average is slightly lower, being Δ*V_th_* variable from +13% to +22%. When the failure modes do not correspond, i.e., cases 4.5, 4.6 and 4.7, the shear resistance given by Equation (1) still overestimates the numerical ones with differences variable from +16.1% to +26.0% for the FEM and from +26.6% to +34.8% for the DMEM.

Finally, for the only case of flexural failure predicted by all models (case 4.8), the agreement of both FEM and DMEM predictions with Equation (2) is very good (Δ*V_th_* is only −7% for the FEM and −3% for the DMEM).

Figure 14 shows the capacity curves provided by the two software for the case *σ_0_* = 0.3 MPa (Figure 14a,b) and 0.6 MPa (Figure 14c). Once again, there is a perfect correspondence between the two approaches in terms of stiffness in the elastic branches. It is worth noting (especially in Figure 14b) that, when the fracture energy in tension increases, the capacity curves of the FEM are able to attain higher ultimate displacements. Conversely, a lower influence of *G_t_* on the ultimate displacement is observed for the DMEM. More in general, Figure 14 highlights that, for most cases, the FEM evaluates a larger ductility in comparison with the DMEM. 

## 5. Discussion on the Shape Factor b Value by Comparing Numerical and Theoretical Shear Capacities

The results reported in Section 4 show that, in most cases, the analytical shear resistances provided by Equation (1) overestimate the predictions given by both the numerical models. One possible reason is the choice of the shape factor *b* that is assumed equal to 1 in this equation, i.e., equal to the examined wall ratio λ = *B*/*H* = 1, as suggested by the Commentary to the Italian code [27].

**Figure 15:**
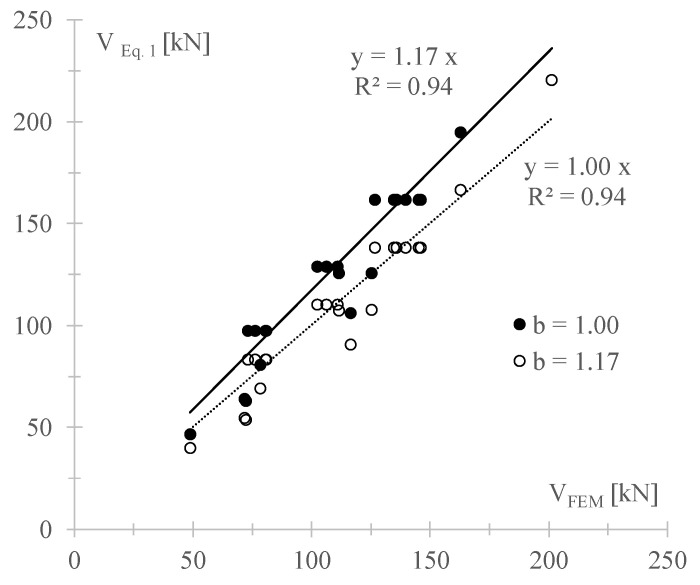
Theoretical predictions of the shear resistance provided by Equation (1) vs. FEM numerical values.

To better investigate the effect of this factor on the in-plane wall response, the numerical shear capacities provided by the FEM are compared, in Figure 15, with the predictions given by Equation (1) for two values of *b*: (1) *b* = 1 as herein assumed, and (2) the value of *b* that best fits the numerical results, i.e., *b* = 1.17, which allows the arranging of the analytical and numerical results along the bisector.

**Figure 16:**
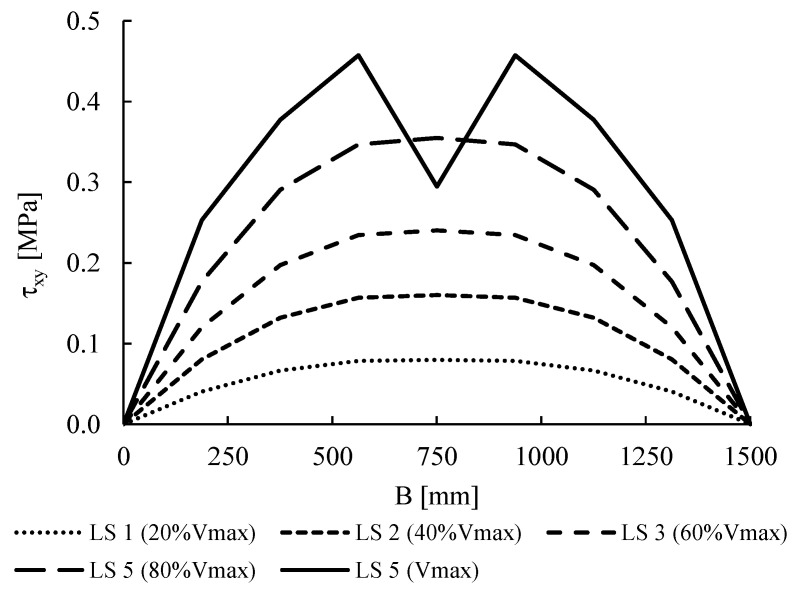
Shear stress distribution along the middle cross-section of the wall for the case 1.6 at different load steps (LS).

The choice of a value other than one for *b,* for squat walls is justified both by the literature [38,39], which individuate values of *b* ranging in 1.15–1.50 even for squat walls, and by the real shear stress distribution observable in the FEM along the middle cross-section of the wall. This distribution, plotted in Figure 16 for the case 1.6 of Group A (*f_c_* = 3 MPa, *f_t_* = 0.15 MPa, σ_0_ = 0.6 MPa), shows, indeed, a parabolic trend for any load step (LS), corresponding to a maximum-to-average stress ratio of about 1.5, independently on the compression stress and on the level of horizontal force. This means that not only at failure is the distribution of shear stress has a parabolic trend, but also that, in general, the assumption of constant shear stresses in the squat wall is not realistic and, thus, the assumption of *b* = 1 in Equation (1) could lead to unsafe predictions. On the contrary, assuming *b* = 1.5, exactly equal to the maximum-to-average stress ratio, could lead to over conservative predictions of the numerical capacity, since the best fitting is obtained in the case of *b* = 1.17.

Clearly, further numerical inquires, also adopting a micro-modelling approach, and experimental tests focussed on this topic are still required to assess a reliable value of the shape factor *b*. However, it can be concluded that the shear resistance provided by Equation (1) for squat walls can be, thus, considered reliable into predicting good numerical results if the coefficient *b* is assumed greater than 1. Note that both models assume a homogenized approach for masonry, which is only able to catch the diagonal shear failure and not the sliding shear failure that is typical of regular masonry walls. However, as suggested by the Commentary to the Italian code and confirmed by previous studies [11], Equation (1) can provide reliable predictions of experimental results also for regular masonry walls.

## 6. Conclusions

Global failure modes generally occur in masonry buildings when the in-plane shear capacity of the resistant elements is reached. The vulnerability of masonry elements to different in-plane failures depends on the achievable mechanisms, which are influenced by both their mechanical properties and geometrical parameters.

In this paper, the in-plane resistance of masonry walls was investigated in several numerical analyses by taking into account the mechanical properties of masonry (compressive and tensile strengths, fracture energy in compression and tension, shear strength) and two different levels of vertical compression. The numerical analyses were carried out by means of a finite element model (FEM) and a discrete macro-element model (DMEM), implemented in the software DIANA FEA and 3DMacro, respectively. Firstly, the reliability of both numerical models was checked by means of comparisons with some experimental tests available in the literature and assumed as benchmarks.

The numerical results provided by the two modelling approaches are in good agreement in terms of failure loads and modes, while some differences can be observed in the load-displacement curves, especially in the non-linear field. In fact, for most cases, the FEM evaluates a larger ductility in comparison with the DMEM.

The numerical results also show that both the compressive and tensile strengths of masonry have a significant influence on the in-plane resistance of the walls, with a higher effect provided by the compression strength. In particular, as the compression strength increases the wall capacity also increases, but less than proportionally. According to the FEM, the shear strength increases, indeed, from +55% to +127%, when the compressive strength is between two and four times higher than the minimum value adopted in the analysis. The shear capacity also increases with the tensile strength according to a less-than-proportional trend and less significantly than that observed with *f_c_*. According to the FEM, in the case of σ_0_ = 0.3 MPa, the shear strength increases by about 40% when *f_t_* increases about four-fold, while for higher compression stress, i.e., σ_0_ = 0.6 MPa, the strength increase is higher, i.e., about 62% for the same variation of *f_t_*. Moreover, in most examined cases and for both models, the increasing of *f_t_* also increases the ultimate displacement of the wall and makes the capacity curves less stepped.

The numerical analyses also prove an effect of the fracture energy in tension on the wall ductility, while a slight influence is observed in terms of shear capacity. In fact, when the fracture energy in tension varies in the fixed range, the strength capacity increases only until 15%, according to the FEM. Finally, the fracture energy in compression does not imply any influence on the behaviour of the masonry walls in terms of both strength and ductility.

Additionally, the numerical resistances obtained by means of the FEM and DMEM are compared with the theoretical formulations proposed by the Italian Code for both flexural and shear failure modes. Regarding the latter one, only the formulation usually provided for irregular/rubble masonry walls was investigated, since it seems more suitable to be compared with the numerical models that are both based on a homogenized approach. Despite the simplification of the theoretical formulations, the comparisons are very good in most cases and it can be noted that the theoretical shear capacity is, on average, greater than the numerical one by approximately 20%. Such a discrepancy can be ascribable to the choice of the shape factor value *b,* suggested by the code formulation, which is related to the shape ratio of the wall. To reduce this discrepancy, the factor *b* should be assumed greater than one, even for squat walls, as also highlighted by other studies. Thus, a proposal for the value of the factor *b* based on the numerical results is done, even if further numerical inquires, also adopting a micro-modelling approach, and experimental tests focussed on this topic are required to confirm the reliability of such a proposal.

Finally, the good agreement attained by the numerical results with the experimental ones, related to tests on walls made of regular masonry, demonstrate that the used macro-modelling approaches can also provide reliable predictions for these types of walls, though these models are only able to predict the diagonal shear (DS) failure, which, nonetheless, is typical of irregular masonry walls. This allows the generalization the results provided by the FEM and DMEM to any type of masonry.

Future works will be focused on the investigation of the behaviour of masonry walls characterized by varying slenderness and on the use of micro-modelling approaches to catch differences between the macro-models proposed in this paper.

## Figures and Tables

**Table 1:** Mechanical properties of masonry used in the DIANA FEM and 3DMacro DMEM.

Parameter			FEM	DMEM
Young’s modulus	*E*	[MPa]	1700	1943
Poisson’s ratio	ν	-	0.20	0.20
Compressive strength	*f_c_*	[MPa]	6.20	6.20
Tensile strength	*f_t_*	[MPa]	0.25	0.25
Compressive fracture energy	*G_c_*	[N/mm]	10	-
Tensile fracture energy	*G_t_*	[N/mm]	0.012	-
Compressive ductility	β*_c_*	-	-	4.6
Tensile ductility	β*_t_*	-	-	3.3
Shear modulus	*G*	[MPa]	-	816
Shear strength	τ_0_	[MPa]	-	0.17
Shear strain capacity	γ*_u_*	[%]	-	0.5
Pre-compression stress	σ_0_	[MPa]	0.6	0.6

**Table 2:** Comparison between experimental, numerical and analytical values of the shear resistance.

Case	Experimental [kN]	Numerical Shear Resistance [kN]		Theoretical Resistance [kN]	
		FEM	DMEM	Equation (1) DS	Equation (2) F
Slender wall	70.5	68.5	68.2	76.8	66.5
Squat wall	81.0	88.1	79.4	85.4	98.5

Underlined values: minimum analytical resistances.

**Table 3:** Mechanical properties used in the sensitivity analyses for Group A.

Group A										
Case	σ_0_	*f_t_*	*f_c_*	*E*	*w*	*G_t_*	*G_c_*	τ_0_	ε*_tr_*	ε*_cr_*
	[MPa]	[MPa]	[MPa]	[MPa]	[kN/m^3^]	[N/mm]	[N/mm]	[MPa]	[%]	[%]
1.1	0.30	0.05	1.50	1000	18	0.005	3.98	0.03	0.15	3.90
**1.2**		**0.15**	**3.00**	**1500**		**0.011**	**7.50**	**0.10**	**0.11**	**3.74**
**1.3**		**0.23**	**4.50**	**1800**		**0.014**	**10.58**	**0.15**	**0.10**	**3.57**
1.4		0.30	6.00	2400		0.017	13.20	0.20	0.09	3.36
1.5	0.60	0.05	1.50	1000	18	0.005	3.98	0.03	0.15	3.90
1.6		0.15	3.00	1500		0.011	7.50	0.10	0.11	3.74
**1.7**		**0.23**	**4.50**	**1800**		**0.014**	**10.58**	**0.15**	**0.10**	**3.57**
**1.8**		**0.30**	**6.00**	**2400**		**0.017**	**13.20**	**0.20**	**0.09**	**3.36**

σ_0_: pre-compression stress, *f_t_*: tensile strength, *f_c_*: compressive strength, *E*: Young’s modulus, *w:* specific weight, *G_t_*: tensile fracture energy, *G_c_*: compressive fracture energy, τ_0_: shear strength, ε*_tr_*: ultimate strains in tension, ε*_cr_*: ultimate strains in compression. Bold values: reference cases that are repeated in the tables of the other groups.

**Table 4:** Mechanical properties used in the sensitivity analyses for Group B.

Group B										
Case	σ_0_	*f_t_*	*f_c_*	*E*	*w*	*G_t_*	*G_c_*	τ_0_	ε*_tr_*	ε*_cr_*
	[MPa]	[MPa]	[MPa]	[MPa]	[kN/m^3^]	[N/mm]	[N/mm]	[MPa]	[%]	[%]
2.1	0.30	0.08	3.0	1500	18	0.007	7.50	0.05	0.13	3.74
**2.2 = 1.2**		**0.15**				**0.011**		**0.10**	**0.11**	
2.3		0.30				0.017		0.20	0.10	
2.4	0.30	0.11	4.5	1800	18	0.009	10.6	0.075	0.12	3.57
**2.5 = 1.3**		**0.23**				**0.014**		**0.15**	**0.10**	
2.6		0.45				0.023		0.30	0.10	
2.7	0.60	0.11	4.5	1800	18	0.009	10.6	0.075	0.12	3.57
**2.8 = 1.7**		**0.23**				**0.014**		**0.15**	**0.10**	
2.9		0.45				0.023		0.30	0.10	
2.10	0.60	0.15	6.0	2400	18	0.011	13.2	0.10	0.11	3.36
**2.11 = 1.8**		**0.30**				**0.017**		**0.20**	**0.09**	
2.12		0.45				0.023		0.30	0.09	

σ_0_: pre-compression stress, *f_t_*: tensile strength, *f_c_*: compressive strength, *E*: Young’s modulus, *w:* specific weight, *G_t_*: tensile fracture energy, *G_c_*: compressive fracture energy, τ_0_: shear strength, ε*_tr_*: ultimate strains in tension, ε*_cr_*: ultimate strains in compression. Bold values: reference cases that are repeated in the tables of the other groups.

**Table 5:** Mechanical properties used in the sensitivity analyses for Group C.

Group C										
Case	σ_0_	*f_t_*	*f_c_*	*E*	*w*	*G_t_*	*G_c_*	τ_0_	ε*_tr_*	ε*_cr_*
	[MPa]	[MPa]	[MPa]	[MPa]	[kN/m^3^]	[N/mm]	[N/mm]	[MPa]	[%]	[%]
3.1	0.30	0.15	3.00	1500	18	0.011	2.0	0.10	0.11	1.14
3.2							4.0			2.07
**3.3 = 1.2**							**7.5**			**3.74**
3.4	0.30	0.23	4.50	1800	18	0.014	4.0	0.15	0.10	1.50
3.5							7.5			2.61
**3.6 = 1.3**							**10.6**			**3.57**
3.7	0.60	0.23	4.50	1800	18	0.014	4.0	0.15	0.10	1.50
3.8							7.5			2.61
**3.9 = 1.7**							**10.6**			**3.57**

σ_0_: pre-compression stress, *f_t_*: tensile strength, *f_c_*: compressive strength, *E*: Young’s modulus, *w:* specific weight, *G_t_*: tensile fracture energy, *G_c_*: compressive fracture energy, τ_0_: shear strength, ε*_tr_*: ultimate strains in tension, ε*_cr_*: ultimate strains in compression. Bold values: reference cases that are repeated in the tables of the other groups.

**Table 6:** Mechanical properties used in the sensitivity analyses for Group D.

Group D										
Case	σ_0_	*f_t_*	*f_c_*	*E*	*w*	*G_t_*	*G_c_*	τ_0_	ε*_tr_*	ε*_cr_*
	[MPa]	[MPa]	[MPa]	[MPa]	[kN/m^3^]	[N/mm]	[N/mm]	[MPa]	[%]	[%]
4.1	0.30	0.15	3.00	1500	18.00	0.003	7.5	0.10	0.04	3.74
4.2						0.005			0.06	
**4.3 = 1.2**						**0.011**			**0.11**	
4.4						0.028			0.27	
4.5	0.30	0.23	4.50	1800	18.00	0.005	10.6	0.15	0.04	3.57
**4.6 = 1.3**						**0.014**			**0.10**	
4.7						0.028			0.19	
4.8						0.050			0.33	
4.9	0.60	0.23	4.50	1800	18.00	0.005	10.6	0.15	0.04	3.57
**4.10 = 1.7**						**0.014**			**0.10**	
4.11						0.028			0.19	
4.12						0.050			0.33	

σ_0_: pre-compression stress, *f_t_*: tensile strength, *f_c_*: compressive strength, *E*: Young’s modulus, *w:* specific weight, *G_t_*: tensile fracture energy, *G_c_*: compressive fracture energy, τ_0_: shear strength, ε*_tr_*: ultimate strains in tension, ε*_cr_*: ultimate strains in compression. Bold values: reference cases that are repeated in the tables of the other groups.

**Table 7:** Shear resistances of Group A.

Case	σ_0_ [MPa]	*f_c_* [MPa]	*f_t_* [MPa]	FEM			DMEM			DMEM vs. FEM	Theor. Resistance		Theor. vs. FEM	Theor. vs. DMEM
				*V*	FM	Δ*V*	*V*	FM	Δ*V*	Δ*V_num_*	*V*_F_ Equation (2)	*V*_DS_ Equation (1)	Δ*V_th_*	Δ*V_th_*
				[kN]		[%]	[kN]		[%]	[%]	[kN]	[kN]	[%]	[%]
1.1	0.30	1.50	0.05	48.9	DS	-	46.6	DS	-	−4.7	86.0	46.7	−4.5	+0.3
1.2		3.00	0.15	80.7	DS	+65.0	82.1	DS	+76.3	1.8	99.3	97.4	+20.7	+18.6
1.3		4.50	0.23	106.2	F	+117.2	99.3	F	+113.2	−6.5	103.7	128.9	−2.4	+4.4
1.4		6.00	0.30	108.1	F	+121.0	122.6	F	+163.1	13.4	105.9	159.1	−2.0	−13.6
1.5	0.60	1.50	0.05	71.8	DS	-	65.3	DS	-	−9.0	119.1	63.9	−11.0	−2.2
1.6		3.00	0.15	111.5	DS	+55.4	117.6	DS	+80.1	5.5	172.1	125.8	+12.8	+6.9
1.7		4.50	0.23	134.8	DS	+87.9	138.7	DS	+112.4	2.9	189.7	161.6	+19.8	+16.5
1.8		6.00	0.30	162.8	DS	+126.9	176.3	DS	+170.0	8.3	198.5	194.9	+19.7	+10.5

DS = diagonal shear failure, F = flexural failure. Underlined values: minimum value between *V*_F_ and *V*_DS._

**Table 8:** Shear resistances of Group B.

Case	σ_0_ [MPa]	*f_t_* [MPa]	*f_c_* [MPa]	FEM			DMEM			DMEM vs. FEM	Theor. Resistance		Theor. vs. FEM	Theor. vs. DMEM
				*V*	FM	Δ*V*	*V*	FM	Δ*V*	Δ*V_num_*	*V*_F_ Equation (2)	*V*_DS_ Equation (1)	Δ*V_th_*	Δ*V_th_*
				[kN]		[%]	[kN]		[%]	[%]	[kN]	[kN]	[%]	[%]
2.1	0.30	0.08	3.0	72.4	DS	-	61.3	DS	-	−15.4	99.3	62.9	−13.2	+2.6
2.2		0.15		80.7	DS	+11.4	82.1	DS	+34.0	+1.8	99.3	97.3	+20.6	+18.5
2.3		0.30		102.9	F	+42.1	114.1	F	+86.2	+10.9	99.3	159.1	−3.5	−13.0
2.4	0.30	0.11	4.5	78.5	DS	-	72.3	DS	-	−7.8	103.7	80.8	+3.0	+11.7
2.5		0.23		106.2	DS	+35.4	99.3	DS	+37.3	−6.5	103.7	128.9	−2.4	+4.4
2.6		0.45		108.8	F	+38.7	144.6	F	+99.9	+32.9	103.7	217.9	−4.7	−28.3
2.7	0.60	0.11	4.5	116.4	DS	-	100.7	DS	-	−13.6	189.7	106.2	−8.8	+5.5
2.8		0.23		134.8	DS	+15.8	138.7	DS	+37.8	+2.9	189.7	161.6	+19.8	+16.5
2.9		0.45		190.8	F	+63.8	188.1	F	+86.9	−1.4	189.7	257.8	−0.6	+0.9
2.10	0.60	0.15	6.0	125.2	DS	-	117.6	DS	-	−6.1	198.5	125.8	+0.5	+7.0
2.11		0.30		162.8	DS	+30.0	176.3	DS	+50.0	+8.3	198.5	194.9	+19.7	+10.5
2.12		0.45		201.3	F	+60.7	188.1	F	+60.0	−6.5	198.5	257.8	−1.4	+5.6

DS = diagonal shear failure, F = flexural failure. Underlined values: minimum value between *V*_F_ and *V*_DS._

**Table 9:** Shear resistances of Group C.

Case	σ_0_ [MPa]	*G_c_* [N/mm]	*f_c_* [MPa]	*f_t_* [MPa]	FEM			DMEM			DMEM vs. FEM	Theor. Resistance		Theor. vs. FEM	Theor. vs. DMEM
					*V*	FM	Δ*V*	*V*	FM	Δ*V*	Δ*V_num_*	*V*_F_. Equation (2)	*V*_DS_ Equation (1)	Δ*V_th_*	Δ*V_th_*
					[kN]		[%]	[kN]		[%]	[%]	[kN]	[kN]	[%]	[%]
3.1	0.30	2.0	3.0	0.15	80.7	DS	-	82.1	DS	-	+1.8	99.3	97.4	+20.7	+18.6
3.2		4.0				DS	0		DS	0	+1.8				
3.3		7.5				DS	0		DS	0	+1.8				
3.4	0.30	4.0	4.5	0.23	106.5	DS	-	99.3	DS	-	−6.7	103.7	128.9	+21.0	+30.0
3.5		7.5			106.4	DS	0		DS	0	−6.7			+21.0	
3.6		10.6			106.2	DS	0		DS	0	−6.5			+21.3	
3.7	0.60	4.0	4.5	0.23	135.9	DS	-	138.7	DS	-	+2.1	189.7	161.6	+18.9	+16.5
3.8		7.5			139.7	DS	+3		DS	0	−0.7			+15.7	
3.9		10.6			134.8	DS	−1		DS	0	+2.9			+19.8	

DS = diagonal shear failure, F = flexural failure. Underlined values: minimum value between *V*_F_ and *V*_DS._

**Table 10:** Shear resistances of Group D.

Case	σ_0_ [MPa]	*G_t_* [N/mm]	*f_c_* [MPa]	*f_t_* [MPa]	FEM			DMEM			DMEM vs. FEM	Theor. Resistance		Theor. vs. FEM	Theor. vs. DMEM
					*V*	FM	Δ*V*	*V*	FM	Δ*V*	Δ*V_num_*	*V*_F_ Equation (2)	*V*_DS_ Equation (1)	Δ*V_th_*	Δ*V_th_*
					[kN]		[%]	[kN]		[%]	[%]	[kN]	[kN]	[%]	[%]
4.1	0.30	0.003	3.0	0.15	73.2	DS	-	79.7	DS	-	+8.8	99.3	97.4	+33.0	+22.2
4.2		0.005			76.1	DS	+3.9	80.9	DS	+1.5	+6.4			+28.1	+20.4
4.3		0.011			80.7	DS	+10.2	82.1	DS	+3.1	+1.8			+20.7	+18.6
4.4		0.028			80.9	DS	+10.5	85.5	DS	+7.3	+5.6			+20.3	+13.9
4.5	0.30	0.005	4.5	0.23	102.3	DS	-	95.6	DS	-	−6.5	103.7	128.9	+26.0	+34.8
4.6		0.014			106.2	DS	+3.8	99.3	DS	+3.8	−6.5			+21.3	+29.8
4.7		0.028			111.0	DS	+8.5	101.8	DS	+6.5	−8.3			+16.1	+26.6
4.8		0.050			111.2	F	+8.7	106.7	F	+11.6	−4.1			−6.8	−2.8
4.9	0.60	0.005	4.5	0.23	126.8	DS	-	135.5	DS	-	+6.9	189.7	161.6	+27.4	+19.2
4.10		0.014			134.8	DS	+6.3	138.7	DS	+2.4	+2.9			+19.8	+16.5
4.11		0.028			145.1	DS	+14.5	141.7	DS	+4.6	−2.4			+11.3	+14.0
4.12		0.050			146.1	DS	+15.2	143.4	DS	+5.8	−1.9			+10.6	+12.7

DS = diagonal shear failure, F = flexural failure. Underlined values: minimum value between *V*_F_ and *V*_DS_.

## Data Availability

Data is contained within the article.
